# Analysis of complete blood count and derived inflammatory indicators for bipolar disorder patients with different states

**DOI:** 10.3389/fpsyt.2023.1219151

**Published:** 2023-07-05

**Authors:** Fangming Xu, Xiaobo Wang, Tianle Zhang, Tingting Xie, Xiao Xin, Yaxuan Zhao, Yumei Wang

**Affiliations:** ^1^Department of Psychiatry, the First Hospital of Hebei Medical University, Shijiazhuang, Hebei Province, China; ^2^Hebei Clinical Research Center for Mental Disorders and Institute of Mental Health, Shijiazhuang, Hebei Province, China; ^3^Mental Health Center, Hebei Medical University and Hebei Technical Innovation Center for Mental Health Assessment and Intervention, Shijiazhuang, Hebei Province, China; ^4^Hebei Key Laboratory of Brain Science and Psychiatric-Psychologic Disease, Shijiazhuang, Hebei Province, China; ^5^Hebei Brain Ageing and Cognitive Neuroscience Laboratory, Shijiazhuang, Hebei Province, China

**Keywords:** bipolar disorder, different states of bipolar disorder, predictors, inflammatory indicators, complete blood count

## Abstract

**Purposes:**

This study aimed to analyze the predictive ability of the complete blood count and derived inflammatory indicators for BD patients with different states to identify potential biomarkers.

**Methods:**

We collected the data of BD in-patients from January 2021 to March 2023. The complete blood count and derived inflammatory indicators were computed by univariate analysis, logistic regression analysis, and receiver operating characteristic (ROC) curve analysis.

**Results:**

In terms of BD patients, the levels of MON (p<0.0001), hs-CRP (*p* = 0.018), and NLR (*p* = 0.002) were independent risk factors in logistic regression analysis, as well as the cut-off values were 0.405 10^9^/L, 2.600 mg/L, and 2.321, respectively. Regarding BD-M patients, the levels of MON (*p*<0.0001), hs-CRP (*p* = 0.012), and NLR (*p* = 0.002) were predictors in logistic regression analysis, and the cut-off values were, respectively, 0.505 10^9^/L, 2.600 mg/L, and 2.620. Additionally, the levels of NLR (*p* = 0.006) and MHR (*p*<0.0001) were important indicators for BD-D and the cut-off values were 1.735 and 0.487, respectively. Furthermore, our findings showed that the level of MON (*p* = 0.001) was related to BD-mixed and the cut-off value was 0.340 10^9^/L. Notably, MON+hs-CRP + NLR, MON+hs-CRP + NLR, and NLR + MHR had the highest diagnostic accuracy to predict BD, BD-M, and BD-D patients, respectively.

**Conclusion:**

Our findings showed that distinct inflammatory indicators were closely associated with BD and its different states. Additionally, we also identified their cut-off values and optimal combined predictive indicators in different states of BD, helping us improve diagnostic accuracy and better assess them to manage early targeted interventions.

## Introduction

Bipolar disorder (BD), which affects 2–3% of the general population (1), is a serious mental disease characterized by the presence of recurring (hypo)mania or depression phases (2), which brings a great burden on families and societies (3). BD is divided into three types: type I patients have at least one manic episode with or without major depressive episodes, type II patients have at least one hypomanic episode and one major depressive episode, and cyclothymic disorder (4). BD patients often have functional disabilities, reduced life quality, and even decreased life expectancy (5). Increasing evidence has been demonstrated that BD patients have almost 30 times suicide risk than healthy people, and high rates of comorbidity (6, 7), such as cardiovascular disease, diabetes mellitus, and chronic obstructive pulmonary disease (8, 9).

Although the etiopathogenesis of BD remains poorly understood, numerous studies have widely reported that the immune and inflammatory systems may be crucial contributors to the onset and progression of this life-threatening illness (10, 11). Wei (12) found that the system inflammation response index (SIRI), neutrophil/high-density lipoprotein(HDL) ratio (NHR), and monocyte/HDL ratio (MHR) were predictors for the BD-M group and MHR was a predictor for the BD-D group. Misiak (13) discovered that the levels of interleukin-8 (IL-8), monocyte-chemoattractant protein-1 (MCP-1), eotaxin-1 and interferon-γ-induced protein 10 (IP-10) were higher in BD patients than in healthy controls. Özdin (14) compared the neutrophil to lymphocyte ratio (NLR), platelet to lymphocyte ratio (PLR), and monocyte to lymphocyte ratio (MLR) values in the manic and euthymic phases of the same patients and identified NLR and PLR as state markers and MLR as a trait marker of BD. Kirlioglu (15) investigated the relationship between inflammatory and metabolic markers and newly diagnosed BD but failed to find evidence supporting high-sensitive C-reactive protein and homocysteine as markers in newly diagnosed BD.

However, the relationship between inflammation factors and BD is still debated because some articles lack healthy people as the control group. As we know, few studies pay attention to the differences in admission laboratory indicators and optimal combined predictive indicators in patients with distinct types of BD. The study aims to investigate the inflammation predictors of BD subtypes compared with healthy people. At the same time, in this study, we not only used a single factor to predict the development of diseases but also used the combined method of multiple risk factors to improve the sensitivity of disease prediction, trying to improve the prediction efficiency through the combined method of multiple factors.

## Patients and methods

### Ethics approval and consent to participate

This retrospective study was approved by the Institutional Review Board of our hospital (20220908) before collecting data. There was no need to obtain informed consent forms from patients because this was a retrospective study.

### Patients

A retrospective analysis of all patients with BD between January 2021 and March 2023 in our hospital. Patients with BD, including BD manic episodes (BD-M), patients with BD depressive episodes (BD-D), and patients with BD mixed episodes (BD-mixed), as well as healthy subjects were considered as the BD group, the BD-M group, the BD-D group, the BD-mixed group, and the healthy group (HG), respectively.

Blood samples were taken within the same time frame (6.00–8.00 a.m.) among these groups. Regarding the BD patients, we collected the first blood samples that were taken on the second day after admission. The inclusion criteria were as follows: (1) patients 18–65 years old, (2) patients with BD in the acute phase based on the International Statistical Classification of Diseases and Related Health Problems 10th Revision (ICD-10) diagnostic criteria, and (3) patients with a first episode or who have not been treated for at least 2 weeks. The exclusion criteria were: (1) patients with other mental diseases, such as schizophrenia, (2) patients with diseases affecting inflammatory markers, such as acute inflammatory diseases, metabolic syndromes (e.g., infections, blood disorders, autoimmune diseases, diabetes, and heart failure), and (3) patients with incomplete data. To be better compared with the BD-M group, BD-D group, and BD-mixed group, data from 102 healthy subjects with age, gender, and BMI matched to the BD group were included as controls. Healthy subjects were recruited if they met the following inclusion criteria: (1) adult patients (between 18 and 65 years old), (2) patients without mental diseases, such as schizophrenia, BD, or depressive disorder, (3) patients without diseases affecting inflammatory markers such as acute inflammatory diseases, metabolic syndromes (e.g., infections, blood disorders, autoimmune diseases, diabetes, heart failure), and (4) completed data.

The demographic data and admission laboratory examinations of patients were collected in this study. The demographic data included age, gender, and BMI. Admission laboratory indicators covered white blood cell (WBC, 10^9^/L), neutrophil (NEU, 10^9^/L), lymphocyte (LYM, 10^9^/L), monocyte (MON, 10^9^/L), red blood cell (RBC, 10^12^/L), hemoglobin (HGB, g/L), platelet (PLT, 10^9^/L), hypersensitive C-reactive protein (hs-CRP, mg/L), Neutrophil-to-lymphocyte ratio (NLR), Monocyte-to-lymphocyte ratio (MLR), Platelet-to-lymphocyte ratio (PLR), Neutrophil-to-HDL ratio (NHR), lymphocyte-to-HDL ratio (LHR), Monocyte-to-HDL ratio (MHR), Platelet-to-lymphocyte ratio (PHR), systemic immune-inflammation index (SII), and system inflammation response index (SIRI). The derived inflammatory markers of the complete blood count are calculated as follows:

NLR = neutrophils count/lymphocytes count;

MLR = monocytes count/lymphocytes count；

PLR = platelets count/lymphocytes count；

NHR = neutrophils count/HDL count；

LHR = lymphocyte count/HDL count；

MHR = monocytes count/HDL count；

PHR = platelets count/HDL count；

SII = (platelets count × neutrophils count)/lymphocytes count；

SIRI = (monocyte count × neutrophils count)/lymphocytes count.

### Statistics

We utilized SPSS (version 25.0 SPSS Inc., Chicago, IL) and regarded *p* < 0.05 as statistical significance. Regarding continuous variables, if data met normality criteria, all measurement data were presented as the mean ± SD (standard deviation) using the t-test, but if not, the Mann–Whitney U test was used to perform statistical analysis between groups. For count data, the Chi-square test was used. Furthermore, to identify the best predictors of BD, we used binary logistic regression analysis to detect its independent predictors. Additionally, receiver operator characteristic (ROC) curve analysis was used to identify the cut-off values for continuous variables and the area under the ROC curve (AUC) was used to determine the diagnostic ability, ranging from 0 to 100%, with more area meaning better ability. We choose the cut-off values for continuous variables by the maximum Youden index (sensitivity+specificity-1) in the ROC curve analysis.

## Results

**Figure 1:**
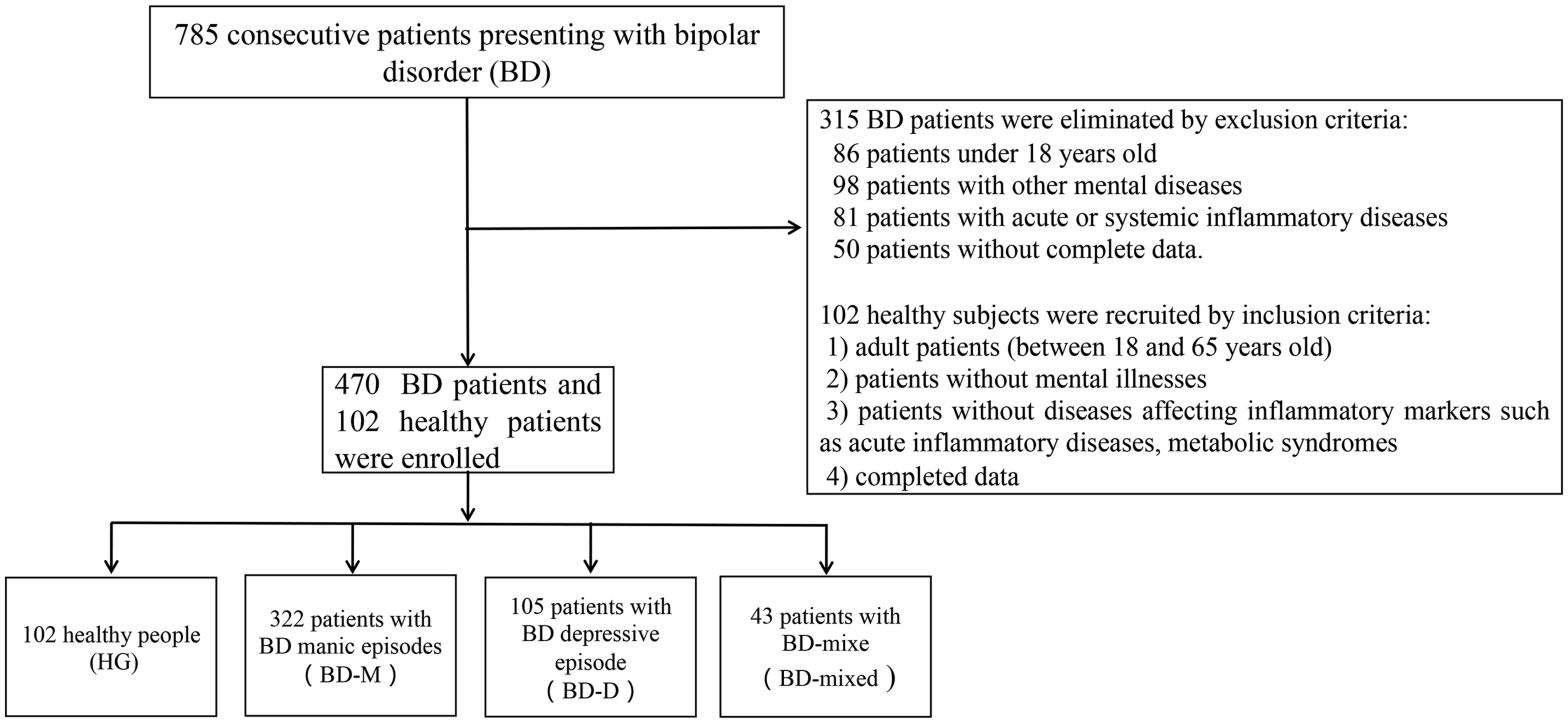
Flow diagram of included patients.

We collected a total of 785 BD patients who presented and were assessed for eligibility in this study. Finally, we collected a total of 470 patients due to the exclusion criteria, including 322 patients with BD manic episodes (BD-M), 105 patients with BD depressive episodes (BD-D), 43 patients with BD mixed episodes (BD-mixed), as well as 102 healthy subjects as the healthy group (HG), as shown in Figure 1.

### Comparison between bipolar disorder or its different states and healthy group by univariate analysis

Regarding the comparison between BD and HG, we discovered that WBC (*p* < 0.0001), NEU (*p* < 0.0001), MON (*p* < 0.0001), RBC(*p* = 0.005), hs-CRP (*p* = 0.010), NLR (*p* < 0.0001), MLR (*p* < 0.0001), NHR (*p* < 0.0001), MHR (*p* < 0.0001), SII (*p* = 0.004), and SIRI (*p* < 0.0001) were involved in the risk of BD (Table 1). In terms of comparison between BD-M and HG, WBC (*p* < 0.0001), NEU (*p* < 0.0001), MON (*p* < 0.0001), RBC (*p* = 0.003), HGB (*p* = 0.048), hs-CRP (*p* < 0.0001), NLR (*p* < 0.0001), MLR (*p* < 0.0001), NHR (*p* < 0.0001), MHR (*p* < 0.0001), SII (*p* = 0.001), and SIRI (*p* < 0.0001) were found to be associated with the risk of BD-M (Table 2). As for comparison between BD-D and HG, we found that MON (*p* = 0.002), RBC (*p* = 0.025), NLR (*p* = 0.030), MLR (*p* < 0.0001), NHR (*p* = 0.020), MHR (*p* = 0.002), and SIRI (*p* = 0.001) were related to BD-D compared with HG, as shown in Table 3. Furthermore, Table 4 indicated that WBC (*p* = 0.002), NEU (*p* = 0.030), MON (*p* < 0.0001), MLR (*p* = 0.001), MHR (*p* = 0.003), and SIRI (*p* < 0.0001) were linked to BD-mixed compared with HG.

**Table 1:** Possible factors may be associated with healthy cases and bipolar disorder patients.

*Characteristics*	*HG (n = 102)*	*BD (n = 470)*	*p*
Age(years)	37.00(30.00–43.00)	34.00(27.00–47.00)	0.354
Gender			0.239
male (*n*, %)	38(37.3%)	205(43.6%)	
female (*n,* %)	64(62.7%)	265(56.4%)	
Body mass index (kg/m^2^)	23.88(22.26–26.73)	24.45(22.09–27.39)	0.640
WBC (10^9^/L)	5.60(5.00–6.55)	6.50(5.30–8.20)	<0.0001
NEU (10^9^/L)	3.20(2.60–3.80)	3.80(2.80–5.13)	<0.0001
LYM (10^9^/L)	1.90(1.60–2.40)	1.90(1.50–2.40)	0.605
MON (10^9^/L)	0.40(0.30–0.50)	0.50(0.40–0.63)	<0.0001
RBC (10^12^/L)	4.61 ± 0.42	4.45 ± 0.50	0.005
HGB(g/L)	136.00(127.75–149.25)	134.00(122.75–146.00)	0.071
PLT (10^9^/L)	248.50(210.00–287.50)	237.00(202.00–274.00)	0.168
hs-CRP (mg/L)	1.10(0.65–1.81)	1.46(0.58–3.84)	0.010
NLR	1.58(1.31–2.02)	1.89(1.38–2.90)	<0.0001
MLR	0.20(0.16–0.24)	0.25(0.20–0.36)	<0.0001
PLR	126.92(102.02–152.54)	124.19(95.49–158.78)	0.747
NHR	2.54(1.91–3.36)	3.32(2.25–4.62)	<0.0001
LHR	1.63(1.23–1.94)	1.56(1.19–2.17)	0.755
MHR	0.31(0.23–0.42)	0.41(0.30–0.57)	<0.0001
PHR	198.09(166.20–254.38)	200.36(157.98–242.78)	0.513
SII	386.56(294.37–506.79)	456.32(312.94–713.04)	0.004
SIRI	0.61(0.43–0.82)	0.94(0.58–1.60)	<0.0001

HG, Healthy group; BD, bipolar disorder; WBC, white blood cell; NEU, neutrophil; LYM, lymphocyte; MON, monocyte; RBC, red blood cell; HGB, hemoglobin; PLT, platelet; hs-CRP, hypersensitive C-reactive protein; NLR, Neutrophil-to-lymphocyte ratio; MLR, Monocyte-to-lymphocyte ratio; PLR, Platelet-to-lymphocyte ratio; NHR, Neutrophil-to-HDL ratio; LHR, lymphocyte-to-HDL ratio; MHR, Monocyte-to-HDL ratio; PHR, Platelet-to-lymphocyte ratio; SII, systemic immune-inflammation index; SIRI, system inflammation response index.

**Table 2:** Possible factors may be associated with healthy cases and bipolar disorder manic episodes.

*Characteristics*	*HG (n = 102)*	*BD-M (n = 322)*	*p*
Age(years)	37.00(30.00–43.00)	34.00(28.00–45.00)	0.279
Gender			0.403
male (*n*, %)	38(37.3%)	135(41.9%)	
female (*n*, %)	64(62.7%)	187(58.1%)	
Body mass index (kg/m^2^)	23.88(22.26–26.73)	24.67(22.45–27.68)	0.324
WBC (10^9^/L)	5.60(5.00–6.55)	6.90(5.48–8.40)	<0.0001
NEU (10^9^/L)	3.20(2.60–3.80)	3.90(2.98–5.36)	<0.0001
LYM (10^9^/L)	1.90(1.60–2.40)	1.90(1.50–2.48)	0.637
MON (10^9^/L)	0.40(0.30–0.50)	0.5(0.40–0.70)	<0.0001
RBC (10^12^/L)	4.61 ± 0.42	4.44 ± 0.50	0.003
HGB(g/L)	136.00(127.75–149.25)	134.00(121–146)	0.048
PLT (10^9^/L)	248.50(210.00–287.50)	237.00(203.75–278.25)	0.235
hs-CRP (mg/L)	1.10(0.65–1.18)	1.73(0.71–4.45)	<0.0001
NLR	1.58(1.31–2.02)	1.93(1.42–3.09)	<0.0001
MLR	0.20(0.16–0.24)	0.27(0.2–0.38)	<0.0001
PLR	126.92(102.02–152.54)	124.77(94.48–223.26)	0.896
NHR	2.54(1.91–3.36)	3.57(2.36–4.78)	<0.0001
LHR	1.63(1.23–1.94)	1.55(1.18–2.24)	0.855
MHR	0.31(0.23–0.42)	0.45(0.32–0.58)	<0.0001
PHR	198.09(166.20–254.38)	202.22(159.13–242.60)	0.632
SII	386.56(294.37–506.79)	473.80(316.83–748.79)	0.001
SIRI	0.61(0.43–0.82)	1.04(0.60–1.89)	<0.0001

HG, Healthy group; BD-M, bipolar disorder manic episodes; WBC, white blood cell; NEU, neutrophil; LYM, lymphocyte; MON, monocyte; RBC, red blood cell; HGB, hemoglobin; PLT, platelet; hs-CRP, hypersensitive C-reactive protein; NLR, Neutrophil-to-lymphocyte ratio; MLR, Monocyte-to-lymphocyte ratio; PLR, Platelet-to-lymphocyte ratio; NHR, Neutrophil-to-HDL ratio; LHR, lymphocyte-to-HDL ratio; MHR, Monocyte-to-HDL ratio; PHR, Platelet-to-lymphocyte ratio; SII, systemic immune-inflammation index; SIRI, system inflammation response index.

**Table 3:** Possible factors may be associated with healthy cases and bipolar disorder depressive episodes.

*Characteristics*	*HG (n = 102)*	*BD-D (n = 105)*	*p*
Age(years)	37.00(30.00–43.00)	37.00(24.50–53.00)	0.937
Gender			
male (*n*, %)	38(37.3%)	50(47.6%)	0.132
female (*n*, %)	64(62.7%)	55(52.4%)	
Body mass index (kg/m^2^)	23.88(22.26–26.73)	24.01(21.28–26.76)	0.372
WBC (10^9^/L)	5.60(5.00–6.55)	5.90(4.85–7.25)	0.346
NEU (10^9^/L)	3.20(2.60–3.80)	3.36(2.61–4.30)	0.145
LYM (10^9^/L)	1.90(1.60–2.40)	1.90(1.50–2.20)	0.266
MON (10^9^/L)	0.40(0.30–0.50)	0.40(0.33–0.57)	0.002
RBC (10^12^/L)	4.61 ± 0.42	4.46 ± 0.53	0.025
HGB(g/L)	136.90 ± 15.75	134.36 ± 15.04	0.239
PLT (10^9^/L)	248.50(210.00–287.50)	235.00(198.50–274.00)	0.132
hs-CRP (mg/L)	1.10(0.65–1.18)	1.00(0.38–2.17)	0.524
NLR	1.58(1.31–2.02)	1.86(1.30–2.36)	0.030
MLR	0.20(0.16–0.24)	0.24(0.19–0.30)	<0.0001
PLR	126.92(102.02–152.54)	125.79(102.42–155.13)	0.964
NHR	2.54(1.91–3.36)	2.84(2.06–4.24)	0.020
LHR	1.63(1.23–1.94)	1.60(1.25–2.09)	0.602
MHR	0.31(0.23–0.42)	0.36(0.27–0.55)	0.002
PHR	198.09(166.20–254.38)	200.75(154.74–250.98)	0.798
SII	386.56(294.37–506.79)	420.43(302.12–622.07)	0.247
SIRI	0.61(0.43–0.82)	0.78(0.50–1.15)	0.001

HG, Healthy group; BD-D, bipolar disorder depressive episodes; WBC, white blood cell; NEU, neutrophil; LYM, lymphocyte; MON, monocyte; RBC, red blood cell; HGB, hemoglobin; PLT, platelet; hs-CRP, hypersensitive C-reactive protein; NLR, Neutrophil-to-lymphocyte ratio; MLR, Monocyte-to-lymphocyte ratio; PLR, Platelet-to-lymphocyte ratio; NHR, Neutrophil-to-HDL ratio; LHR, lymphocyte-to-HDL ratio; MHR, Monocyte-to-HDL ratio; PHR, Platelet-to-lymphocyte ratio; SII, systemic immune-inflammation index; SIRI, system inflammation response index.

**Table 4:** Possible factors may be associated with healthy cases and bipolar disorder with mixed features.

*Characteristics*	*HG (n = 102)*	*BD with mixed features (n = 43)*	*p*
Age(years)	37.00(30.00–43.00)	33.00(29.00–45.00)	0.245
Gender			0.299
male (*n*, %)	38(37.3%)	20(46.5%)	
female (*n*, %)	64(62.7%)	23(53.5%)	
Body mass index (kg/m^2^)	23.88(22.26–26.73)	24.41(21.88–26.83)	0.883
WBC (10^9^/L)	5.60(5.00–6.55)	6.70(5.50–8.90)	0.002
NEU (10^9^/L)	3.20(2.60–3.80)	3.40(2.81–5.90)	0.030
LYM (10^9^/L)	1.90(1.60–2.40)	2.00(1.70–2.38)	0.375
MON (10^9^/L)	0.40(0.30–0.50)	0.50(0.40–0.60)	<0.0001
RBC (10^12^/L)	4.61 ± 0.42	4.53 ± 0.44	0.366
HGB(g/L)	136.00(127.75–149.25)	137.00(124.00–149.00)	0.797
PLT (10^9^/L)	248.50(210.00–287.50)	244.00(203.00–267.00)	0.497
hs-CRP (mg/L)	1.10(0.65–1.18)	0.90(0.48–2.37)	0.962
NLR	1.58(1.31–2.02)	1.70(1.33–2.93)	0.115
MLR	0.20(0.16–0.24)	0.24(0.20–0.31)	0.001
PLR	126.92(102.02–152.54)	110.24(90.34–150.00)	0.112
NHR	2.54(1.91–3.36)	2.90(1.96–5.06)	0.087
LHR	1.63(1.23–1.94)	1.64(1.20–2.15)	0.825
MHR	0.31(0.23–0.42)	0.38(0.30–0.51)	0.003
PHR	198.09(166.20–254.38)	188.32(147.11–223.53)	0.116
SII	386.56(294.37–506.79)	438.89(312.08–758.43)	0.197
SIRI	0.61(0.43–0.82)	0.73(0.65–1.32)	<0.0001

HG, Healthy group; BD with mixed features, bipolar disorder with mixed features; WBC, white blood cell; NEU, neutrophil; LYM, lymphocyte; MON, monocyte; RBC, red blood cell; HGB, hemoglobin; PLT, platelet; hs-CRP, hypersensitive C-reactive protein; NLR, Neutrophil-to-lymphocyte ratio; MLR, Monocyte-to-lymphocyte ratio; PLR, Platelet-to-lymphocyte ratio; NHR, Neutrophil-to-HDL ratio; LHR, lymphocyte-to-HDL ratio; MHR, Monocyte-to-HDL ratio; PHR, Platelet-to-lymphocyte ratio; SII, systemic immune-inflammation index; SIRI, system inflammation response index.

### Independent risk factors and cut-off values of bipolar disorder and its different states by logistic regression analysis and ROC curve analysis

**Figure 2:**
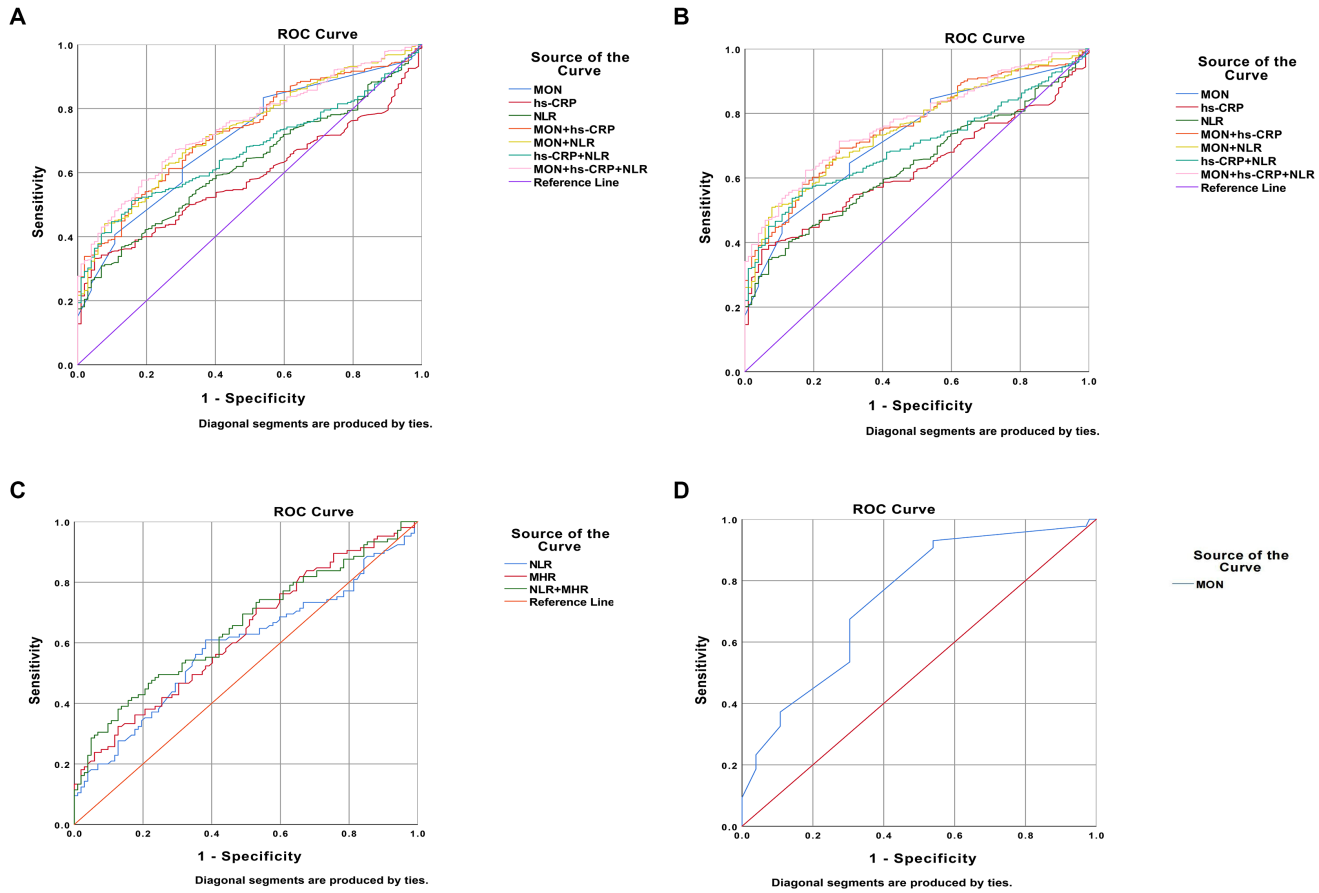
Predictors of bipolar disorder (BD) patients in receiver operating characteristic curve analysis. **(A)**. BD patients vs. healthy people; **(B)**. BD manic patients vs. healthy people; **(C)**. BD depressive patients vs. healthy people; **(D)**. patients with BD mixed episodes vs. healthy people.

Regarding BD patients, the level of MON [*p*<0.0001, OR = 161.257, 95%CI (24.065, 1080.585)], hs-CRP [*p* = 0.018, OR = 1.190, 95%CI (1.031, 1.374)], and NLR [*p* = 0.002, OR = 2.819, 95%CI (1.484, 5.354)] were independent risk factors (Table 5). ROC curve analysis showed that the level of MON [p<0.0001, AUC area = 0.704, 95%CI (0.653,0.754)], hs-CRP [*p* = 0.010, AUC area = 0.582, 95%CI (0.533, 0.631)], NLR [*p*<0.0001, AUC area = 0.622, 95%CI (0.571, 0.673)], MON+ hs-CRP [p<0.0001, AUC area = 0.727, 95%CI (0.680, 0.774)], MON+NLR [p<0.0001, AUC area = 0.732, 95%CI (0.684, 0.779)], hs-CRP + NLR [*p*<0.0001, AUC area = 0.667, 95%CI (0.620, 0.713)], and MON+hs-CRP + NLR [*p*<0.0001, AUC area = 0.746, 95%CI (0.701, 0.790)] were predictors of BD. Additionally, the cut-off values of MON, hs-CRP, and NLR to predict BD were 0.405 10^9^/L, 2.600 mg/L, and 2.321, respectively (Table 6; Figure 2).

**Table 5:** Predictors for bipolar disorder in different states by multivariate analysis.

	*B*	*S.E*	*p value*	*Odds ratio*	*95% CI*	
					*Lower limit*	*Upper limit*
*HG vs BD*						
MON	5.083	0.971	<0.0001	161.257	24.065	1080.585
hs-CRP	0.174	0.073	0.018	1.190	1.031	1.374
NLR	1.036	0.327	0.002	2.819	1.484	5.354
Constant	2.151	1.168	0.065	8.593		
*HG vs BD-M*						
MON	5.375	1.011	<0.0001	215.916	29.737	1567.718
hs-CRP	0.211	0.084	0.012	1.235	1.048	1.457
NLR	1.022	0.335	0.002	2.779	1.440	5.360
Constant	1.520	1.291	0.239	4.571		
*HG vs BD-D*						
NLR	0.544	0.200	0.006	1.723	1.165	2.549
MHR	4.208	1.066	<0.0001	67.212	8.322	542.860
Constant	3.244	1.495	0.030	25.632		
*HG vs BD with mixed features*						
MON	5.454	1.718	0.001	233.805	8.065	6778.348
Constant	−4.308	0.822	<0.0001	0.013		

HG, Healthy group; BD, bipolar disorder; BD-M, bipolar disorder manic episodes; BD-D, bipolar disorder depressive episodes; BD with mixed features, bipolar disorder with mixed features; MON, monocyte; hs-CRP, hypersensitive C-reactive protein; NLR, Neutrophil-to-lymphocyte ratio; MHR, Monocyte-to-HDL ratio.

**Table 6:** ROC curve analysis and cut-off values of bipolar disorder in different states.

*Variables*	*Area*	*p-value*	*95%CI*		*Cut-off value*
			*Lower limit*	*Upper limit*	
*HG vs BD*					
MON	0.704	<0.0001	0.653	0.754	0.405
hs-CRP	0.582	0.010	0.533	0.631	2.600
NLR	0.622	<0.0001	0.571	0.673	2.321
MON+ hs-CRP	0.727	<0.0001	0.680	0.774	NA
MON+NLR	0.732	<0.0001	0.684	0.779	NA
hs-CRP + NLR	0.667	<0.0001	0.620	0.713	NA
MON+ hs-CRP + NLR	0.746	<0.0001	0.701	0.790	NA
*HG vs BD-M*					
MON	0.726	<0.0001	0.675	0.777	0.505
hs-CRP	0.628	<0.0001	0.575	0.681	2.600
NLR	0.639	<0.0001	0.585	0.693	2.620
MON+ hs-CRP	0.757	<0.0001	0.710	0.805	NA
MON+ NLR	0.756	<0.0001	0.708	0.803	NA
hs-CRP+ NLR	0.697	<0.0001	0.648	0.747	NA
MON+ hs-CRP+ NLR	0.775	<0.0001	0.729	0.820	NA
*HG vs BD-D*					
NLR	0.587	0.030	0.509	0.665	1.735
MHR	0.626	0.002	0.551	0.702	0.487
NLR+ MHR	0.652	<0.0001	0.578	0.726	NA
*HG vs BD with mixed features*					
MON	0.740	<0.0001	0.655	0.826	0.340

HG, Healthy group; BD, bipolar disorder; BD-M, bipolar disorder manic episodes; BD-D, bipolar disorder depressive episodes; BD with mixed features, bipolar disorder with mixed features; MON, monocyte; hs-CRP, hypersensitive C-reactive protein; NLR, Neutrophil-to-lymphocyte ratio; MHR, Monocyte-to-HDL ratio.

Interestingly, the predictors of BD-M patients were consistent with the risk factors of BD patients the levels of MON [*p*<0.0001, OR = 215.916, 95%CI (29.737, 1567.718)], hs-CRP [*p* = 0.012, OR = 1.235, 95%CI (1.048, 1.457)], and NLR [*p* = 0.002, OR = 2.779, 95%CI (1.440, 5.360)] (Table 5). ROC curve analysis showed that the level of MON [*p*<0.0001, AUC area = 0.726, 95%CI (0.675, 0.777)], hs-CRP [p<0.0001, AUC area = 0.628, 95%CI (0.575, 0.681)], NLR [p<0.0001, AUC area = 0.639, 95%CI (0.585, 0.693)], MON+ hs-CRP [p<0.0001, AUC area = 0.757, 95%CI (0.710, 0.805)], MON+NLR [*p*<0.0001, AUC area = 0.756, 95%CI (0.708, 0.803)], hs-CRP + NLR [*p*<0.0001, AUC area = 0.697, 95%CI (0.648, 0.747)], and MON+hs-CRP + NLR [*p*<0.0001, AUC area = 0.775, 95%CI (0.729, 0.820)] were predictors of BD-M. Additionally, the cut-off values of MON, hs-CRP, and NLR to predict BD-M were 0.505 10^9^/L, 2.600 mg/L, and 2.620, respectively (Table 6; Figure 2).

In terms of BD-D patients, the level of NLR [*p* = 0.006, OR = 1.723, 95% CI (1.165, 2.549)] and MHR [*p*<0.0001, OR = 67.212, 95% CI (8.322, 542.860)] were independent risk factors (Table 5). ROC curve analysis showed that the level of NLR [*p* = 0.030, AUC area = 0.587, 95%CI (0.509, 0.665)], MHR [*p* = 0.002, AUC area = 0.626, 95%CI (0.551, 0.702)], and NLR + MHR [*p*<0.0001, AUC area = 0.652, 95%CI (0.578, 0.726)] were predictors of BD-D. Additionally, the cut-off values of NLR and MHR to predict BD-D were 1.735 and 0.487 (Table 6; Figure 2).

In addition, our findings showed that the level of MON [*p* = 0.001, OR = 233.805, 95%CI (8.065, 6778.348)] was an independent risk factor for BD-mixed (Table 5). ROC curve analysis showed that the level of MON [p<0.0001, AUC area = 0.740, 95%CI (0.655, 0.826)] was a predictor of BD-mixed. Additionally, the cut-off value of MON to predict BD-mixed was 0.340 10^9^/L (Table 6; Figure 2).

## Discussion

Recently, growing attention has been paid to the hot topic of the relationship between inflammation and BD. Wei (12) considered the SIRI, NHR, and MHR to be predictors to differentiate BD-M patients from healthy people and regarded the MHR as a predictor for differentiating BD-D patients from healthy people, which was inconsistent with the findings of Dadouli (16) that MLR was the only risk factor for BD-M patients and no indicator was found to be related to BD-D compared with the control group. Ongoing evidence has investigated the role of inflammation in BD, yet their true and concrete relationship is not completely understood (10–13). Additionally, most related studies focused on the comparison between BD patients and BD-M patients or between BD-D patients due to a lack of data from healthy individuals. As we know, few studies have assessed the inflammatory indicators between BD patients and healthy individuals based on subgroups of BD.

Our findings showed that numerous immunological factors were associated with BD and its different states by univariate analysis. In terms of BD patients, the levels of MON, hs-CRP, and NLR were independent risk factors in logistic regression analysis, as well as the cut-off values were 0.405 10^9^/L, 2.600 mg/L, and 2.321, respectively. Regarding BD-M patients, the levels of MON, hs-CRP, and NLR were predictors in logistic regression analysis, and the cut-off values were, respectively, 0.505 10^9^/L, 2.600 mg/L, and 2.620. Additionally, the level of NLR and MHR were close indicators for BD-D and the cut-off values were 1.735 and 0.487, respectively. Furthermore, our findings showed that the level of MON was related to BD-mixed and the cut-off value was 0.340 10^9^/L. Notably, MON+hs-CRP + NLR, MON+hs-CRP + NLR, and NLR + MHR had the highest diagnostic accuracy to predict the BD, BD-M, and BD-D patients, respectively.

Monocytes are the most crucial cells for the secretion of pro-inflammatory and pro-oxidant cytokines, which are essential elements of the innate immune reaction against pathogens (17). Increased monocyte counts and alterations have been demonstrated to be associated with affective disorders (18, 19). In the present study, monocyte counts were found to be independent predictors of BD and BD-M patients compared with healthy subjects. Furthermore, we also identified the cut-off value of MON to predict BD subtypes. Wei (12) found no significantly elevated monocyte counts, which was inconsistent with our results. The potential reason that can explain the discrepancy with Wei (12) is that the BMI was not matched between control cases and BD patients in his study, which can influence the inflammation indexes. While increased MON was only used to differentiate between patients with BD-M and healthy controls in the study of Dadouli (16), which was partially similar to our findings. These discrepancies may be attributed to the different time frames in which cases and control blood samples were collected, or race differences in the research of Dadouli, which had great effects on the difference in our results. Notably, another important finding was that the cut-off value of MON to predict BD-M patients was higher than that of BD patients, implying that when monocyte counts were higher than 0.405 10^9^/L or 0.505 10^9^/L, patients were more likely to suffer from BD or BD-M, respectively.

C-reactive protein (CRP) is a pentamer acute-phase protein produced by the liver and secreted into the blood. CRP plays an important role in the innate immune system, rising rapidly in response to infection and inflammation, declining sharply after the acute phase. Fernandes (20) found that the level of CRP was higher in BD patients than in the health group regardless of their mood states, while the CRP levels of BD-M patients were much higher than in other states. In addition, it has been reported that the hs-CRP level of BD-M patients was significantly higher than in the health group before treatment, and the hs-CRP level was significantly reduced after treatment (21). These results were partially similar to our findings. In our study, hs-CRP was found to be an independent predictor of BD and BD-M patients compared with healthy cases. Notably, the same cut-off values of hs-CRP were identified to predict BD and BD-M patients.

NLR, PLR, MLR, NHR, LHR, MHR, PHR, SII, and SIRI index are cost-effective and simple laboratory parameters, implying a balance of systemic inflammation in many diseases (22–25). Our results showed that compared to HG, NLR was higher in BD patients, BD-M patients, and BD-D patients, it was considered a key predictor for BD patients, BD-M patients, and BD-D patients, which was consistent with Dadouli’s findings. However, regarding patients with BD-D, MHR was found to be a risk factor compared with healthy individuals, and its cut-off value was demonstrated. Wei (12) found that the MHR and NHR were independent predictors for identifying BD-M patients from the healthy group in ROC curve analysis, while the MHR was an independent predictor for differentiating BD-D patients from the healthy group, which is consistent with our results.

Other blood indexes, such as lymphocyte number, PLR, or SII, have been proven to play crucial roles in many diseases (26, 27), which are related to BD in recent studies (14, 15, 28). In our study, these indicators were significantly higher in BD patients or BD subtype groups than in healthy people, whereas they were demonstrated to have a negative relationship with BD in ROC curve analysis. A large sample and multi-center study are needed to assess their value in the prediction of BD.

Furthermore, we tried to find the combined predictors with the highest diagnostic accuracy in BD patients with different states compared with healthy subjects using inflammatory indicators. Regarding BD patients, MON+hs-CRP + NLR was found to have the highest diagnostic accuracy (AUC area = 0.746) to predict these patients, while MON+hs-CRP + NLR (AUC area = 0.775) was the best combined predictor of BD-M patients. In terms of BD-D patients, NLR + MHR (AUC area = 0.652) was the optimal indicator. Pointing out the best-combined predictors and their cut-off values can effectively and maximally help us distinguish different stages of BD patients as early as possible. We still need a larger sample to further verify these results.

However, some limitations should not be neglected. First, we divided BD patients into groups based on their mood episodes regardless of the severity of their symptoms, so the predictors of the severity of symptoms were not analyzed in this retrospective study. Second, some confounders, such as smoking and diet, may influence the inflammation indicators. Third, we did not collect other potential biomarkers regarding inflammation in our study due to the retrospective study.

In conclusion, we found various inflammatory indicators associated with BD and its subtypes. Regarding BD patients and BD-M patients, the levels of MON, hs-CRP, and NLR were independent risk factors in logistic regression analysis, while the levels of NLR and MHR were independent risk factors for BD-D. However, MON was an independent risk factor for BD-mixed. Furthermore, our findings identified the cut-off values and optimally combined indicators to predict BD, BD-M, BD-D, and BD-mixed, which help us better assess them to manage early targeted interventions.

## Data availability statement

The original contributions presented in the study are included in the article/supplementary material, further inquiries can be directed to the corresponding author.

## Ethics statement

This study involving humans was reviewed and approved by the Institutional Review Board of the First hospital of Hebei Medical University (20220908). Write informed consent was not required in accordance with local and national regulations.

## Author contributions

FX and XW was responsible for study concept and writing the article. TX, TZ, XX, and YZ were responsible for screened the abstracts and reviewed the article. YW was responsible for reviewing and writing the article. All authors contributed to the article and approved the submitted version.

## Funding

This work was supported by the STI2030-Major Projects (2021ZD0200700) and National Natural Science Foundation of China Project (No. 81771463).

## Conflict of interest

The authors declare that the research was conducted in the absence of any commercial or financial relationships that could be construed as a potential conflict of interest.

## Publisher’s note

All claims expressed in this article are solely those of the authors and do not necessarily represent those of their affiliated organizations, or those of the publisher, the editors and the reviewers. Any product that may be evaluated in this article, or claim that may be made by its manufacturer, is not guaranteed or endorsed by the publisher.
